# Extraction of causal relations based on SBEL and BERT model

**DOI:** 10.1093/database/baab005

**Published:** 2021-02-18

**Authors:** Yifan Shao, Haoru Li, Jinghang Gu, Longhua Qian, Guodong Zhou

**Affiliations:** School of Computer Science and Technology, Soochow University, Suzhou, Jiangsu Province, China, 215006; School of Computer Science and Technology, Soochow University, Suzhou, Jiangsu Province, China, 215006; Department of Chinese & Bilingual Studies, The Hong Kong Polytechnic University, Hong Kong, China, 999077; School of Computer Science and Technology, Soochow University, Suzhou, Jiangsu Province, China, 215006; School of Computer Science and Technology, Soochow University, Suzhou, Jiangsu Province, China, 215006

## Abstract

Extraction of causal relations between biomedical entities in the form of Biological Expression Language (BEL) poses a new challenge to the community of biomedical text mining due to the complexity of BEL statements. We propose a simplified form of BEL statements [Simplified Biological Expression Language (SBEL)] to facilitate BEL extraction and employ BERT (Bidirectional Encoder Representation from Transformers) to improve the performance of causal relation extraction (RE). On the one hand, BEL statement extraction is transformed into the extraction of an intermediate form—SBEL statement, which is then further decomposed into two subtasks: entity RE and entity function detection. On the other hand, we use a powerful pretrained BERT model to both extract entity relations and detect entity functions, aiming to improve the performance of two subtasks. Entity relations and functions are then combined into SBEL statements and finally merged into BEL statements. Experimental results on the BioCreative-V Track 4 corpus demonstrate that our method achieves the state-of-the-art performance in BEL statement extraction with *F*1 scores of 54.8% in Stage 2 evaluation and of 30.1% in Stage 1 evaluation, respectively.

**Database URL**: https://github.com/grapeff/SBEL_datasets

## Introduction

Biomedical entity relation extraction (RE) identifies the semantic relationships between biomedical entities (such as genes, proteins, chemicals, diseases, biological processes and so on), such as protein–protein interactions (1–3), drug-to-drug interactions (4, 5) and relations between chemicals and proteins (7–9). It is of great significance to the construction of biomedical knowledge bases, precision medicine and new drug discovery as well. Majority of these entity relations represent a single interaction or regulatory relation between two biomedical entities and cannot fully reflect more complex causal relationship involving multiple biomedical entities, and therefore, the form and scope of knowledge they can express are quite restricted. The BioCreative-V community organized a shared task4 (http://www.biocreative.org/tasks/biocreative-v/track-4-bel-task/) to extract the causal relation between biomedical entities from the literature in the form of Biological Expression Language (BEL (6); http://www.openbel.org/), which is appropriate for both machine processing and human reading. BEL can express not only causal relations between entities, but also functions around entities. This form of representation has great capacity to express rich domain-specific knowledge; it, however, poses new challenges to biomedical text mining.

There are roughly three existing strategies for tackling automatic extraction of BEL statements: rule-based methods, cross-task ones and intra-task ones. The rule-based method, such as that by Ravikumar *et al.* (10, 11), introduces a rule-based semantic analyzer to perform the BEL extraction task. Due to the high complexity of BEL statements, it obtains an *F*1 value of 21.29%; for cross-task methods, the NCU-IISR system by Lai *et al.* (12) first uses the biomedical semantic role labeling technology to parse a sentence into the predicate–argument structure and then converts it to BEL statement, achieving an *F*1 measure of 32.08%; Choi *et al.* (13) propose an event-based extraction method and further use the reference resolution technique to identify more entities and thus more BEL statements, which achieves the performance with *F*1 of 35%. The reason for the low scores in cross-task methods is that information loss is unavoidable in transferring instances between different tasks, and furthermore, the training corpus provided by the BEL task is not used at all. Following the success of deep learning on many NLP (Natural Language Processing) tasks as well as RE, Liu *et al.* (14) propose an intra-task method to directly train a deep-learning model on the BEL training corpus. They cast the BEL extraction task as a combination of two fundamental subtasks, RE and function detection (FD), and further use attention-based BiLSTM models to extract relations and functions that are further combined into BEL statements. Through the confidence threshold filtering of detected entity functions, their final BEL statement performance reaches the *F*1 value of 46.9%. Generally speaking, BEL statement extraction remains a challenging task with the *F*1 value below 50%. The reasons are 2-fold: one is that the training set used for RE and FD is relatively small due to the loss when it is converted from complex BEL statements and the other is that the overall performance of RE and FD in the biomedical domain is not sufficiently high.

To address the aforementioned issues, we follow the path of Liu *et al.* (14) and further introduce the concept of Simplified Biological Expression Language (SBEL) statements, thus transform the BEL statement extraction into the extraction of SBEL statements, so as to make full use of as many training instances (including relations and functions) as possible. Meanwhile, we employ the powerful BERT model by Devlin *et al.* (15), which has demonstrated the effectiveness of contextualized word representations in fine-tuning a specific task from a pretrained language model.

## Materials and methods

### Dataset preparation

#### Complexity of BEL statements

**Figure 1.**
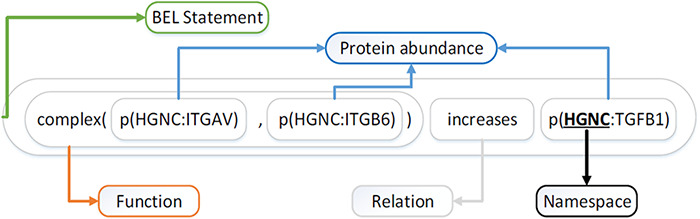
Example of BEL statement.

Selven *et al.* (16) first proposed the BEL in 2011, which is designed to represent the complex causal relationship between biomedical entities in the field of life sciences. BEL is not only editable but also easily readable by humans. Figure 1 illustrates an example of BEL statement ‘complex(p(HGNC:ITGAV), p(HGNC:ITGB6)) increases (HGNC:TGFB1)’.

BEL statements generally consist of Term, Function and Relation (17, 18). Terms or entities contain entity identifiers and entity types, along with their namespaces. Entity types include proteins, chemicals, diseases and biological processes. For example, the term ‘p(HGNC:TGFB1)’ refers to the protein entity (TGBFBI) defined in the HGNC (HUGO Gene Nomenclature Committee) namespace. Function ‘*complex*()’ expresses the combination of protein (ITGAV) and protein (ITGB6). The causal relationship (a predicate) ‘*increases*’ indicates that the combination of the subject (HGNC:ITGAV and HGNC:ITGB6) promotes the abundance of the object (HGNC:TGFB1).

Compared with the conventional RE task with two involved entities and no functions at all, causal RE in BEL statements poses a significant challenge in NLP community. Through our analysis of the BioCreative-V Task 4 corpus, the complexity of BEL statements can be reflected in the following aspects:

- Nested relations: a causal relationship between entities can participate in another causal relationship as a subject or object term. For example, the BEL statement ‘p(MGI:Ins2) increases (act (p(MGI:Akt1)) increases p(MGI:Pde3b,pmod(p)))’ (BEL:200590504) indicates that the protein (MGI:Ins2) can promote the activity of the protein (MGI:Akt1) on the phosphorylation of the protein (MGI:Pde3b).
- Nested functions: an entity function is nested in another function. For example, the BEL statement ‘cat(complex(p(HGNC:ITGA2), p(HGNC:ITGB1))) increases bp(GOBP: “cell fungins”)’ (BEL: 20073926) expresses that the catalysis of combination of two proteins promotes the biological process of cell adhesion.
- Function with multiple entities: a function related to several entities. Take the case of the above BEL statement (BEL:20073926), the combination of two proteins (HGNC: ITGA2 and HGNC:ITGB1) constitutes a function involving multiple entities.
- Self-relations: a kind of special relationship between an entity and itself, where one function of an entity increases/decreases another function of the same entity. For example, the BEL statement ‘p(HGNC:CTNNB1, pmod(p,S,37)) directlyIncreases deg(p(HGNC:CTNNB1))’ (BEL: 20002944) denotes that phosphorylation of the protein (HGNC:CTNNB1) directly leads to its own degradation.
- Multiple relations: One entity may entail various functions, giving rise to multiple relationships between two entities. For example, two BEL statements, ‘p(HGNC:SUMO1) increases cat(p (HGNC:MDM2))’ (BEL:20045200) and ‘p(HGNC: SUMO1) decreases deg(p(HGNC:MDM2))’ (BEL: 20045202), indicate that the protein (HGNC: SUMO1) can not only promote the catalysis of protein (HGNC:MDM2), but also inhibit its degradation.

BEL extraction presents new challenges and opportunities as well for the biological text mining community. The conventional RE (19, 20, 21, 22) in biomedical domain can neither deal with the difficulty of self-relations and multiple relations, nor can it tackle the issue of the nested relations. If the BEL statement is regarded as a kind of semantic representation and its extraction as semantic parsing, then it is confronted with difficult issues like insufficient corpus and the erroneous alignment between entity identifiers and their mentions in text.

#### SBEL statements

Due to the aforementioned complexity of BEL statements and the fact that the proportion of complex statements is relatively low, this paper proposes to use an intermediate form of SBEL statements to extract BEL statements. The basic idea is to transform or discard complex structures in BEL statements, while retaining the relations between two entities as many as possible.

Formally, SBEL statements can be defined as follows:

<SBEL> =: <Subject> <Relation> <Object>

<Subject> =: <Function>(Entity) | <Entity>

<Object> =: <Function>(Entity) | <Entity>

<Entity> =: <DatabaseID>: <EntityID>

<Relation> =: Increases | Decreases

<Function> =: act | cat | pmod | …

where <Subject> and <Object> represent BEL Terms and <Relation> describes the relationship between the subject and the object. A BEL term or an entity can be modified with a <Function>, which represents a specific biological function, and an <Entity> consists of a database identifier and an entity identifier.

In short, an SBEL statement expresses the causal relationship between a subject and an object both with at most one function. Due to its simplicity, an SBEL statement can be further encoded in a quintuple:

<*func1*, entity1, *relation, func2*, entity2>

where func1 and func2 are the corresponding functions to entity1 (subject) and entity2 (object) respectively, and the relation is the one between the subject and the object. The following SBEL example indicates that entity (HGNC:SUMO1) promotes the catalytic activity of entity (HGNC:MDM2):

<*None*, HGNC:SUMO1, *increases, cat*, HGNC:MDM2>

Different from the research in Liu *et al.* (14), this paper considers functions with multiple arguments, mainly the ‘*complex()*’ function that takes a list of arguments. However, emphasized that function ‘*complex()*’ in a BEL statement must be decomposed to multiple single-argument functions in order to generate multiple SBEL statements.

#### Conversion between BEL and SBEL statements

##### Conversion of BEL statements to SBEL statements.

Since a BEL statement has more powerful expressiveness than an SBEL statement, information loss is unavoidable during the conversion from the former to the latter. Our goal is to retain sufficient information of the original BEL statements as much as possible. Specifically, for the complex BEL statements, we perform the following processing steps:

- Nested relations: we select the statement for conversion in nested BEL statements that only contains entities (possibly with functions) as the subject and object, so as to produce more SBEL statements and thus increase training corpus size.
- Nested functions: we pick up the intermediate function of an entity as its function and discard its upper functions to ensure that an entity has at most one function. The assumption here is that the keyword expressing the intermediate function is closest to the entity in the text.
- Function with multiple entities: It is important to note that only the ‘*complex()*’ function has multiple arguments. To obtain as many SBEL statements as possible, ‘*complex()*’ is distributed to each of its entities to form multiple SBEL statements with the other subject or object.
- Self-relations: the BEL statement containing the same entity in the subject and object is discarded, because the current binary RE model cannot deal with self-relations.
- Multiple relations: if there are multiple relationships between two entities, only the first BEL statement is selected for conversion. Since SBEL extraction will ultimately be transformed into the task of binary RE with single label and multiple classes, only one relation type can be retained between a pair of entities.
- Standard conversion: a BEL statement with two entities as the subject and the object with at most one function is directly converted to an SBEL statement.

For example, the following BEL statement (BEL:20073928) involves nested functions with multiple entities:

‘cat(complex(p(HGNC:ITGA2),p(HGNC:ITGB1)))

increases bp(GOBP: “cell adhesion”)’

After the above conversion, two separate SBEL statements are available as follows:

*SBEL1*: <*complex*, HGNC:ITGA2, *increases, None*,

GOBP: ‘cell adhesion’>

*SBEL2*: <*complex*, HGNC:ITGB1, *increases, None*,

GOBP: ‘cell adhesion’>

Obviously, there is some degree of information loss for conversion steps (i), (ii), (iv) and (v). As we can see from the above BEL statement (BEL:20073928), the function ‘*cat()*’ is lost during the conversion from BEL to SBEL.

##### Merging of SBEL statements to BEL statements.

It is much easier to merge SBEL statements to BEL statements than the other way. We can transform an SBEL quintuple into a BEL statement by concatenating the two entities’ functions with the relation, in the form of ‘*func1*(entity1) *relation func2*(entity2)’ (note that the function should be omitted if it is ‘None’). Differently, Ravikumar *et al.* (10, 11) first extract BEL functions and then determine relationships involving functions to complete a BEL statement. In addition to functions in Liu *et al.* (14), more functions like ‘*complex()*’ and ‘*tloc()*’ are included in our SBEL statements, and therefore, when the entity function in an SBEL statement is ‘*complex()*’, the merging of ‘*complex()*’ functions with the same entity should be performed. The merging strategy is as follows:

##### Subject merging.

When the subject function in a SBEL statement is ‘*complex()*’, it should be merged with other SBEL statements with the ‘*complex()*’ function in subject as well as the same predicate and object. The entities in the subjects of these statements constitute a new entity set, which is assigned to the same ‘*complex()*’ function in order to form a new BEL statement.

##### Object merging.

Corresponding to subject merging, when the object function is ‘*complex()*’, SBEL statements with the *complex()* function in subject and the same predicate and object should be merged. Similarly, the entities in their objects constitute a new entity set, and the set with ‘*complex()*’ function forms a new BEL statements.

In the subsection ‘Conversion of BEL statements to SBEL statements’, two separate SBEL statements SBEL1 and SBEL2 are taken as examples. Their functions in the subject are ‘*complex()*’ and their predicates and objects are the same, and therefore, the original BEL statement (BEL:20073928) without ‘*cat()*’ can be obtained by subject merging. Note that the difference between the original statement and the regenerated statement is caused by the conversion from BEL to SBEL, not by the merging of SBEL to BEL since it is intuitive to see that the merging retains all the information in SBEL.

### Dataset statistics

#### Statistics of SBEL statements on the corpus

The corpus provided by BioCreative-V BEL task includes a training set and a test set both in sentences (18). The statistics of sentences, BEL statements, transformed SBEL statements, relations and functions in this corpus from top to bottom, as well as relations and functions in SBEL statements for training and test sets are shown in Table 1 from top to bottom, where relations and functions are further broken down into their minor categories. For comparison, statistics of transformed set by Liu *et al.* (14) is also listed in column 3 and column 5. It can be observed from the table that:

**Table 1.** Statistics on the BC-V BEL task corpus

	Training		Test	
Statistics	Ours	Liu et al. (14)	Ours	Liu et al. (14)
Sentence	6353	6353	105	105
BEL	11066	11066	202	202
SBEL	10097	–	203	–
Relations	10097	9176	203	166
increases	7382	6701	150	121
decreases	2715	2475	53	45
Functions	6476	5226	67	38
act	4637	4163	27	25
deg	119	103	6	6
pmod	712	698	5	3
sec	217	226	4	4
tloc	63	–	5	–
complex	728	–	22	–

- Due to information loss in the conversion from BEL to SBEL, the number of SBEL statements in the training set is less than that of the BEL statements. This is in contrast to the case with the test set where the SBEL statements brought about by the decomposition of ‘*complex()*’ functions outnumber the lost SBEL statements in the conversion from BEL to SBEL.
- The number of binary relations is exactly the same as that of SBEL statements. This is because the SBEL statements with redundant multiple relations have been eliminated. There is one-to-one correspondence between SBEL and binary relations, while Liu *et al.* (14) did not remove redundant statements.
- The number of function instances in the training set exceeds more than half of that of relation instances, while the number of function instances in the test set accounts for less than half of relation instances, indicating that most entities in SBEL statements of the training set have functions, while most entities in the test set do not.

#### Measuring the conversion loss

Although the merging of SBEL to BEL statement is lossless, there is information loss vice versa. We calculate the *P*/*R*/*F*1 scores of reconstructed BEL statements against the original BEL statements to measure the degree of information loss during conversion of BEL to SBEL. Specifically, BEL statements of the training set and test set are first converted into SBEL statements respectively, and then, these SBEL statements are reconstructed back to BEL statements. Using the original BEL statements as the gold standard, the performance scores of the reconstructed BEL statements was evaluated at various levels. The evaluation results are shown in Tables 2 and 3, for the training set and test set respectively, where State(REL) denotes the performance at BEL statement level when considering only relations and ignoring functions, and State(MRG) denotes the performance at BEL statement level when entity functions are combined into their relations.

**Table:** SBEL performance on the BC-V training set

	Training		
Evaluation levels	*P* (%)	*R* (%)	*F*1 (%)
Term	99.68	96.73	98.19
FS	97.47	84.35	90.43
Function	96.34	82.97	89.16
RS	99.92	96.90	98.39
Relation	99.46	93.13	96.19
State(REL)	43.75	36.41	39.74
State(MRG)	89.58	81.59	**85.4**

**Table:** SBEL performance on the BC-V test set

	Testing		
Evaluation levels	*P* (%)	*R* (%)	*F*1 (%)
Term	100.00	97.97	98.97
FS	97.78	84.62	90.72
Function	96.23	83.61	89.47
RS	100.00	97.52	98.75
Relation	100.00	95.54	97.72
State(REL)	53.72	58.56	56.03
State(MRG)	91.10	86.14	**88.55**

It can be observed from the table that:

- The *F*1 scores at BEL statement (MRG) level in both the training set and test set are above 85%, indicating that the loss of extracting BEL by using SBEL as an intermediate form is acceptable. We can also say that complex BEL statements only account for a small number and the loss rate is less than 15 units. The *F*1 score of the test set ($\sim$90%) is higher than that of the training set, meaning lower conversion losses. Furthermore, for both training and test sets, precision scores are higher than recall scores, which means that more false negatives than false positives are made in the conversion process from BEL to SBEL.
- The performance scores of the training set and the test set BEL statement (REL) are generally not high, with the *F*1 scores $\sim$40% and $\sim$56% respectively, dramatically lower than those of BEL statement (MRG). This implies that a large number of entities in BEL statements carry functions, and ignoring these functions will drastically decrease the BEL statement performance. In other words, if we only consider relations between involved entities and ignore their functions, it is absolutely impossible to significantly improve BEL statement performance.
- The performance scores of the training and test sets at other evaluation levels (Term, Function and Relation) are very similar, which indicates that the main difference between two sets is in the combination of relations and functions. Since ignoring function incurs more performance degeneration in the training set ($\sim$45 units of *F*1) than in the test set ($\sim$32 units of *F*1), it can be considered that the influence of functions on BEL statements is stronger in the training set than in the test set.

## Methods

### BEL statement extraction based on SBEL

The basic idea behind BEL extraction based on SBEL is that an intermediate format SBEL is adopted between complex BEL statements and fundamental binary and unary relations. First, BEL statements in the original training corpus are transformed into SBEL statements, which are in turn used to train both RE and FD models. Then, these two models are applied to the test set to predict both relations between entities and entity functions, which are further combined to SBEL statements. Finally, we assemble BEL statements from SBEL statements.

### SBEL statement extraction based on RE and FD

For the extraction of SBEL statements, we follow the similar path as Liu *et al.* (14), i.e. the task is decomposed into two subtasks: extraction of binary relations between entities and detection of entity functions. The difference lies in the statements to be decomposed. We decompose SBEL statements to relations and functions, while in the work by Liu *et al.* (14), it is the original BEL statements to be directly decomposed. Here the procedure follows three stages:

- SBEL statements obtained on the training set are decomposed into relation and function instances. It should be noted that if an entity appears in multiple SBEL statements with different functions, the function that appears for the first time is selected to ensure that one entity has exactly one function.
- Two BERT models are used to train RE and FD (as unary RE) respectively. The difference between RE and FD is that for RE every pair of two entities in the sentence is regarded as a potential instance, while for FD, every entity is regarded as a potential instance.
- Predict binary relationship for each pair of entities on the test set and one function (unary relation) for each entity. If there is a causal relationship between an entity pair, two involved entities, their respective functions and the relation are combined to form a quintuple, i.e. an SBEL statement.

In recent decades, research on RE in biomedical field has made great progress, as in the general domain. In addition to conventional machine learning methods such as SVM (Support Vector Machines) (19) and KNN (K-Nearest Neighbor) (20), deep learning methods such as CNN (Convolutional Neural Network) (4) and RNN (Recurrent Neural Network) (22) also exhibit superior performance. In particular, BERT (15), which is a dominant pretrained language model in recent years, not only greatly improves the performance of binary RE in the general domain, but also performs excellently in the biomedical domain [BioBERT (23)]. Naturally, we use BERT to both extract binary relations and entity functions.

**Figure 2.**
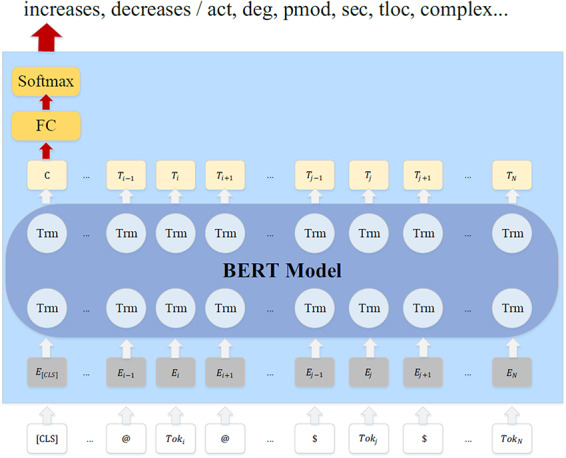
The BERT model for RE/FD.

BERT is a pretrained language model using Transformer (24) as a feature extractor, which converts input sentences or pairs of sentences into hidden vector sequences. Furthermore, BERT uses an MLM (Mask Language Model) (25), which can predict randomly masked words in a sequence, so bidirectional contexts are considered to train word representations. After pretraining, only fine-tuning on a specific task is needed. Figure 2 shows the structural diagram of fine-tuning a sentence-level multi-class classification task (RE and FD) on the BERT model. In the figure, $To{k_i}$ and $To{k_j}$ in the input sequence are referred to the 1st and 2nd entities respectively with their surface names replaced with placeholders. @ and $ are special delimiters to mark the two entities respectively. It needs to be emphasized that we simply mark just one entity with @ when dealing with FD. ${E_1},{E_2} \cdots {E_N}$ denote the input word vectors, ${T_1},{T_2} \cdots {T_N}$ denote the contextual representations from the BERT model. [CLS] is a special token used to output classification label. A fully connected layer (FC) and a softmax layer are stacked on the [CLS] output, in order to get the classification labels for RE and FD separately.

The standard BERT pretraining corpora come from BooksCorpus (26) and English Wikipedia dataset; it may not perform best in biomedical domain. Therefore, we adopt the BioBERT (23) model, which is pretrained on the combination of PubMed abstracts (PubMed) and PubMed Central full-text articles. More important, BioBERT achieves excellent performance in several biomedical text mining tasks including biomedical RE.

### Experimentation

This section first introduces the hyper-parameters of our model, then describes the evaluation datasets and metrics and finally details the experimental results.

### Hyper-parameter setting

We use the BioBERT version ‘biobert-pubmed-v1.1’ as the BERT encoder. The fine-tuning parameters of RE and FD are shown in Table 4.

**Table 4.** Neural network parameters

Parameters	Value
batch_size	4
epoch	3
max_seq_len	100
loss function	categorical_crossentropy
Learning rate	1e-5
optimizer	Adam

### Evaluation datasets

The corpus was provided by the organizer for the BioCreative V BEL task, which contains the training, sample and test sets. There is also a similar task (27) in the 2017 BioCreative VI, which uses the same training set as BC-V, but provides a new test set. However, the new test set is not publicly available, and therefore, we conduct experiments and compare results on the BC-V test set.

### Evaluation metrics

We use standard metrics to evaluate the performance at a certain level, namely, Precision (*P*), Recall (*R*) and *F*1 (f1-measure). Precision refers to the ratio of the number of correct instances to the total number of instances extracted by the system. Recall refers to the ratio of the number of correct instances extracted from the system to the number of gold instances. *F*1 represents the harmonic mean of precision and recall. The three metrics can be defined as follows, where TP, FP and FN mean the numbers of true positives, false positives and false negatives respectively.

(1) \begin{equation*}P = {{TP} \over {TP + FP}}\end{equation*}

 

(2) \begin{equation*}R = {{TP} \over {TP + FN}}\end{equation*}

 

(3) \begin{equation*}F1 = {{2*P*R} \over {P + R}}\end{equation*}

### Experimental results

#### Cross-validation performance of RE and FD on the BC-V training set

Ten-fold cross-validation is performed on the training set, and the results are compared with Liu *et al.* (14), as shown in Table 5. The three involved models are Att-BiLSTM on the training set in Liu *et al.* (14), BERT (14) (we merely trained ourselves BERT models on the training set of Liu *et al.* (14) since they only experiment with Att-BiLSTM models) on the same training set and SBEL-BERT on our training set. Each model is run five times, and its average performance is taken as the final result. The values in the parentheses right to *F*1 scores are the standard deviations of the results of five runs. The highest values of *P*/*R*/*F*1 among the three models in each row are shown in bold typeface. It can be seen from the table that:

**Table 5.** Ten-fold cross-validation performance of RE and FD on the BC-V training set

	Att-BiLSTM (14) (%)			BERT (14) (%)			SBEL-BERT (ours) (%)		
Types	*P*	*R*	*F*1	*P*	*R*	*F*1	*P*	*R*	*F*1
Relations	61.7	60.8	61.3(±1.4)	**72.9**	68.6	**70.5**(±0.3)	72.4	**69.0**	**70.5**(±0.2)
increases	65.1	**73.4**	69.2(±1.4)	**73.5**	69.2	71.1(±1.6)	73.2	70.3	**71.5**(±1.6)
decreases	54.2	40.0	46.0(±2.4)	**71.4**	**67.3**	**68.9**(±2.6)	70.5	65.5	67.6(±2.0)
Functions	53.9	**54.0**	**53.9**(±2.5)	**55.6**	46.5	50.2(±0.5)	55.4	47.0	50.5(±0.9)
act	52.3	**59.8**	**56.0**(±4.1)	56.5	46.4	50.4(±3.7)	**57.6**	47.7	51.4(±4.4)
deg	**58.8**	16.9	26.3(±16.0)	52.6	**41.2**	**43.5**(±11.1)	52.4	40.5	43.0(±13.2)
pmod	**59.5**	32.8	42.3(±5.8)	54.4	**48.0**	49.5(±7.2)	54.6	47.9	**49.8**(±6.2)
sec	51.7	23.1	31.9(±9.2)	55.3	47.5	49.1(±9.0)	**58.4**	**53.2**	**53.3**(±12.0)
tloc	–	–	–	–	–	–	**46.0**	**36.9**	**37.2**(±20.0)
complex	–	–	–	–	–	–	**48.3**	**41.3**	**43.8**(±6.0)

- No matter what kind of corpus is used, the RE performance of BERT models is improved by about 10 units compared with the Att-BiLSTM model, which is much anticipated since BERT is a more powerful model. The increase largely comes from the ‘*decrease*’ relation with fewer instances than the ‘*increase*’ relation, implying that BERT can better alleviate the problem of data sparsity than Att-BiLSTM.
- In terms of FD, BERT has no superiority over Att-BiLSTM as BERT obtains a little lower *F*1 score, which may explain that the original BERT model might not be suitable for FD. However, the precision for ‘*act()*’ function with BERT is much higher than that with Att-BiLSTM. As pointed out in Liu *et al.* (14), the precision of FD plays a critical role when combining functions into relations. The higher precision of FD, the greater the performance contribution of combining functions into relations to form BEL statements.
- Our training set for FD includes two additional types, i.e. ‘*tloc()*’ and ‘*complex()*’, whose performance is generally not high and needs to be improved. In particular, ‘*complex()*’ function involves the assembly of multiple entity functions, so it will have a significant impact on the performance of BEL statements.

#### Performance on the BC-V test set with/without functions

We evaluate our SBEL-BERT model on the BC-V test set with gold entities, known as Stage 2 BEL evaluation. In this case, the whole training set is used to train the models, and the induced models are then applied to the test set. The results at various evaluation levels by three methods are shown in Table 6. Similarly, the highest *P*/*R*/*F*1 scores in each row among three models are highlighted in bold. It can be seen that:

**Table 6.** Performance in stage 2 on the BC-V test set with considering functions

Evaluation levels	Att-BiLSTM (14) (%)			BERT (14) (%)			SBEL-BERT (ours) (%)		
	*P*	*R*	*F*1	*P*	*R*	*F*1	*P*	*R*	*F*1
Term	**99.3**	**95.2**	**97.2**(±0.7)	98.4	80.5	88.6(±1.6)	98.5	90.2	94.2(±1.6)
FS	43.3	45.2	44.3(±2.3)	60.7	38.5	46.9(±1.9)	**80.2**	**52.3**	**63.2**(±1.9)
Function	31.7	36.7	34.0(±2.9)	43.6	31.7	36.5(±1.4)	**60.7**	**39.7**	**47.9**(±2.9)
RS	98.8	**94.4**	**96.5**(±0.7)	**99.8**	87.2	93.0(±1.6)	99.6	92.2	95.8(±1.3)
Relation	66.2	65.4	65.8(±0.8)	**85.1**	64.6	73.4(±1.3)	80.2	**69.3**	**74.3**(±1.9)
State(REL)	45.1	44.8	44.9(±1.0)	**67.0**	46.9	**53.5**(±1.1)	55.6	**49.2**	52.1(±1.0)
State(MRG)	42.5	41.2	41.7(±1.6)	**59.0**	45.1	51.1(±3.3)	**59.0**	**51.3**	**54.8**(±1.8)

- At Relation level, both BERT models perform much better than Att-BiLSTM by a margin of about 8 units in *F*1 score, though the performance on RS level by Att-BiLSTM performs better than BERT due to its loose evaluation. Compared with Liu *et al.* (14), SBEL-BERT on our training set achieves higher recall but lower precision, probably because our training set is bigger than theirs (c.f. Table 1).
- At State(REL) level, both BERT models outperform Att-BiLSTM by about 8 units in *F*1 score due to the significant improvement of RE. However, our SBEL-BERT model does not outperform the BERT (14) model due to significant lower precision. This is a bit different from the cross-validation scenario on the training set where two BERT models perform comparably on RE.
- At Function/FS levels, BERT models consistently outperform the Att-BiLSTM model. The reasons may be 2-fold: Our training set has more function types (including ‘*tloc()*’ and ‘*complex()*’) than that in (14), leading to better precision and recall. The second is that the performance gaps between BERT and Att-BiLSTM on the test set and the 10-fold cross-validation reflect the fact that the distributions of the training and test sets on function instances are quite different as shown in Table 1. Nevertheless, this conclusion is not statistically evident because of the limited number of functions in the test set.
- At State(MRG) level, our model based on BERT and SBEL achieves the best *F*1 score of 54.8, which outperforms the state-of-the-art Att-BiLSTM model. While for BERT (14) the *P*/*R*/*F*1 scores at State(MRG) are lower than those at State(REL) caused by erroneous functions in BEL statements due to low precision in FD, for our SBEL-BERT model, the *P*/*R*/*F*1 scores State(MRG) are higher than those at State(REL) by 2$\sim$3 units, thanks to the high precision in FD.

We also experiment with the case where gold entities on the test set are not provided (defined as Stage 1), and the same approach as Liu *et al.* (14) is used to automatically recognize entity mentions and link them to the corresponding databases. After that, our trained models are applied to entity mentions in the test sentences to identify entity functions and relations between entity pairs. Finally, two entity functions and their relation constitute an SBEL quintuple, which is ultimately transformed into a BEL statement. Table 7 reports the performance by three models on the test set. Likewise, the highest *P/R/F1* scores in each row among three models are highlighted in bold. Other experimental setting is similar to Stage 2.

**Table:** Performance in Stage 1 on the BC-V test set without considering functions

	Att-BiLSTM (14) (%)			BERT (14) (%)			SBEL-BERT (ours) (%)		
Evaluation levels	*P*	*R*	*F*1	*P*	*R*	*F*1	*P*	*R*	*F*1
Term	56.3	63.6	58.6(±0.9)	**65.2**	49.0	55.9(±2.6)	59.7	**60.1**	**59.8**(±0.8)
FS	66.7	23.1	34.3(±1.4)	**76.3**	30.0	42.1(±6.4)	72.7	**51.5**	**59.6**(±4.5)
Function	**36.8**	11.7	17.7(±2.1)	35.3	15.5	20.8(±5.4)	32.9	**26.0**	**28.5**(±3.0)
RS	57.6	67.8	62.3(±1.7)	**80.2**	61.5	69.4(±2.6)	73.0	**71.6**	**72.2**(±1.1)
Relation	27.7	36.6	31.6(±1.8)	**46.2**	33.8	38.8(±1.4)	40.0	**41.2**	**40.4**(±1.0)
State(REL)	16.0	23.3	19.0(±1.2)	**31.2**	22.8	26.2(±0.8)	27.4	**28.5**	**27.8**(±0.7)
State(MRG)	18.7	24.8	21.3(±1.8)	**33.7**	24.8	28.3(±1.4)	29.8	**30.8**	**30.1**(±1.4)

Compared with Stage 2, lower performance in *P*/*R*/*F*1 at all evaluation levels is apparently due to the noise associated with automatic named entity recognition and entity normalization. Interestingly, BERT (14) achieves consistently the highest precision at almost all levels, while our SBEL-BERT obtains the best recall and *F*1 score. This may be due to relatively larger SBEL training set with ‘*tloc()*’ and ‘*complex()*’ functions and more relation instances, leading to better generalization capability at the expense of lower precision for the BERT model.

#### Comparison with other systems

We compare our model with other models on the BC-V BEL test set in Stage 1(the upper part) and Stage 2 (the lower part) evaluation at various levels in Table 8. The four systems compared are (i) rule-based model (10, 11), (ii) event-based model (13), (iii) NCU-IISR (12) and (iv) Att-BiLSTM model (14). The best performance of *F*1 scores for each column is shown in boldface in the table.

As shown in Table 8, in Stage 2, our system achieved the best performance at four evaluation levels (including relation and function levels), particularly at the BEL statement level, where the *F*1 value reaches 54.8%, outperforming other systems by at least 8 units. This demonstrates the efficacy of our model based on SBEL and BERT. In Stage 1, we observe that our system still achieves competitive performance, surpassing other systems except the rule-based one (10, 11).

**Table 8.** Performance comparison with other systems on the BC-V test set in Stage 1 and Stage 2

Systems	T	FS	Fun	RS	Rel	Stat
Rule-based	**62.9**	55.4	**42.6**	**73.3**	**49.2**	**39.2**
Event-based	34.0	10.0	8.6	25.1	41.4	20.2
NCU-IISR	45.0	9.5	2.7	56.7	26.4	19.7
Att-BiLSTM	58.6	34.3	17.7	62.3	31.6	21.3
SBEL-BERT	59.8	**59.6**	28.5	72.2	40.4	30.1
Rule-based	82.4	56.5	30.0	82.4	65.1	25.6
Event-based	54.3	26.1	20.8	61.5	43.7	35.2
NCU-IISR	55.2	–	–	63.5	44.6	33.1
Att-BiLSTM	**97.2**	34.8	26.6	**96.5**	65.8	46.9
SBEL-BERT	94.2	**63.2**	**47.9**	95.8	**74.3**	**54.8**

### Error analysis

We perform error analysis in order to better understand the complexity and difficulty of BC-V BEL extraction task and divide the errors into the following five categories:

- Modeling deficiency
  Due to the nature of SBEL statements, some complex BEL statements involving nested relations, nested functions, with multiple arguments, self-relation and multiple relations, cannot be recognized. They are discarded during the process of being converted to SBEL. It is also impossible to be reconstructed from SBEL. The results in Table 2 suggest that, even if the performance of RE and FD is perfect, i.e. 100%, the *F*1 score at the BEL statement level is less than 90%.
- Misaligned entity mentions
  Aligning entity identifiers in a BEL statement to entity mentions in the corresponding sentence is performed before converting BEL to SBEL. We use the same approach as in Liu *et al.* (14) to entity alignment, which is a fuzzy matching algorithm based on edit distance. In some cases, entity identifiers may be aligned to erroneous entity mentions or may not be aligned at all. For example, for the sentence ‘ClC-3 is activated by Ca(2+)-calmodulin-dependent protein kinase II; however, the magnitude of the Ca(2+)-dependent Cl(-) current was unchanged in the Clcn3(-/-) animals.’ (SEN:10003704) and its BEL statement ‘p(MGI:Camk2a) increases act(p(MGI:Clcn3))’ (BEL:20037754), the entity ‘MGI:Camk2a’ in the BEL statement is misaligned to ‘Ca’ in the sentence, leading to cascading errors to downstream RE and FD.
- Function errors
  The causes of function type errors can be further divided into three subcategories:
  - Ambiguous keywords: some keywords express ambiguous functions. For example, for the sentence ‘In addition, the expression of a S100A13 mutant lacking a sequence novel to this gene product functions as a dominant-negative repressor of IL-1alpha release.’ (SEN:10010034) and its BEL statement ‘p(MGI:S100a13) increases sec(p(MGI:Il1a))’ (BEL: 20060574), the keyword ‘release’ in the sentence can express both ‘*sec()*’ function and ‘*act()*’ function, leading to erroneous prediction of entity function.
  - Apposition interference: apposition structure may interfere FD in some cases. For instance, for the sentence ‘In chow-fed Pctp-/- mice, acyl CoA:cholesterol acyltransferase (Acat) activity was markedly increased, 3-hydroxy-3-methylglutaryl-CoA reductase activity was unchanged, and cholesterol 7alpha-hydroxylase activity was reduced.’ (SEN:10004988) and its BEL statements ‘p(MGI:Pctp) decreases act(p(MGI:Soat1))’ (BEL:20032282) as well as ‘p(MGI:Pctp) increases act(p(MGI:Cyp7a1))’ (BEL: 20032284), two entity mentions ‘acyl CoA: cholesterol acyltransferase’ and its apposition ‘*Acat*’ in the sentence are referred to the same entity with ‘*act()*’ function. We select the closest pair of two entity mentions as the training instance for RE and designate functions to these two mentions without considering apposition structure during training. This may lead to errors in FD in some cases.
  - Lack of domain knowledge: one challenging issue with causal extraction in biomedical domain is that a large number of entity functions can only be inferred by assistance from domain knowledge. For example, for the sentence ‘RSK4 inhibition partially rescued BRAFE600-induced senescence in both TIG3 and TIG3 p16-null.’ (SEN:10037214) and its BEL statement ‘p(HGNC:BRAF, sub(V,600,E)) increases kin(p(HGNC:RPS6KA6))’ (BEL:20079246), we observe that ‘RPS6KA6’ is a kind of kinases with *kin()* function in the HGNC (https://www.genenames.org/) database where protein ‘HGNC:RPS6KA6’ is described as ‘ribosomal protein S6 kinase A6’. However, this knowledge is not used in our model.
- Relation errors
  The errors in RE are mainly caused by the complex sentence pattern expressing causality relationship, which can be further divided into the following three subcategories:
  - Long-distance dependence: Long-distance dependency is always a challenging issue in NLP, though it has been significantly alleviated in BERT. For example, for the sentence ‘A unique inhibitory FcgammaR, FcgammaRIIB, inhibits intracellular signaling upon ligation of IgG-immune complexes, and can suppress inflammation and autoimmunity.’ (SEN:10004778) and its two BEL statements ‘p(MGI:Fcgr2b) decreases’ (BEL:200313762) and ‘p(MGI:Fcgr2b) decreases path(MESHD: Inflammation)’ (BEL:200313761), the entity ‘MGI:Fcgr2b’ is far from entities ‘MESHD: Auto-immune Diseases’ and ‘MESHD:Inflammation’ in the sentence.
  - Negation inversion: Gene knockout technology is often used in biomedical experiments to test the function of a gene and this knockout reverses the direction of causal relationship. For example, for the sentence ‘Following LPS inhalation, alveolar neutrophil levels and lung inflammation in ADAM17-null mice were overall reduced when compared to control mice.’ (SEN: 10007908) and its BEL statement ‘p(MGI:Adam17) increases bp(GOBP: inflammatory response)’ (BEL: 200467681), the sentence indicates that, after the mouse gene ‘MGI:Adam17’ was knocked out, the physiological process of ‘GOBP: inflammatory response’ was decreased, that is, the gene ‘MGI:Adam17’ promotes, not reduces ‘GOBP: inflammatory response.’ This complex semantic inversion sometimes cannot be fully captured by the model.
  - Complex coordinate conjunction: complex coordination structures complicate RE. As an example, the sentence ‘IL-1alpha enhanced and reduced, respectively, the levels of Cx33 and Cx43 mRNA in a time- and dose-dependent manner.’ (SEN: 10009198) entails the BEL statements ‘p(MGI:Il1a) decreases r(MGI:Gja1)’ (BEL:20054682) and ‘p(MGI:Il1a) increases r(MGI:Gja6)’ (BEL:20054684). Two trigger words (‘enhanced’ and ‘reduced’) in the sentence expressing two opposite relation types govern two entities in coordinate structure; however, our model cannot accurately discriminate which trigger word acts on which entity, resulting in RE error for the second BEL statement.
- Annotation errors
  Due to the complexity of causality, a small number of annotation errors is inevitable. To illustrate this, for the sentence ‘In the CFTR-KO mice, the ELF concentration of GSH was decreased (51%) compared with that in WT mice.’ (SEN:10008290) and its BEL statement ‘p(MGI:Cftr) decreases a(CHEBI:glutathione)’ (BEL:20049348), we believe that the gene ‘MGI:Cftr’ has a promotional effect on the chemical ‘CHEBI:glutathione’ since the word ‘null’ is involved in the sentence.

## Conclusion

Following the work by Liu *et al.* (14), we apply the similar idea of decomposing BEL statement extraction into RE and FD subtasks. Differently, an intermediate statement form (SBEL statement) bridges the gap between BEL statements with rich entity functions and relation instances without entity functions. SBEL enhances the expressivity of relations, thus entails more learning instances than the previous one and leaves space for further improvement, though at the expense of losing a small fraction of BEL statements. Meanwhile, we employ the more powerful BERT model than the original Att-BiLSTM model in order to achieve better performance for RE and FD. Ultimately, experimental results on the BioCreative-V Track 4 corpus demonstrate that our method significantly improves the performance of BEL statement extraction. Our system achieves the state-of-the-art results in Stage 2 evaluation with an *F*1 score of 54.8%.

One deficiency is that our system cannot achieve a satisfactory level of performance in FD. Therefore, one direction in future research is how to use a more effective model or incorporate more features to improve the FD performance. On the other hand, we will also explore the joint learning strategy between RE and FD in SBEL statement extraction, aiming to make full use of the dependence between relations and functions.
